# Impact of Prior Influenza and Pneumoccocal Vaccines on Humoral and Cellular Response to SARS-CoV-2 BNT162b2 Vaccination

**DOI:** 10.3390/vaccines9060615

**Published:** 2021-06-08

**Authors:** Vincenzo Puro, Concetta Castilletti, Chiara Agrati, Delia Goletti, Sara Leone, Alessandro Agresta, Eleonora Cimini, Eleonora Tartaglia, Rita Casetti, Francesca Colavita, Silvia Meschi, Giulia Matusali, Daniele Lapa, Saeid Najafi Fard, Alessandra Aiello, Chiara Farrone, Paola Gallì, Maria Rosaria Capobianchi, Giuseppe Ippolito, on behalf of the INMI COVID-19 Vaccine Study Group

**Affiliations:** National Institute for Infectious Diseases Lazzaro Spallanzani, 00149 Rome, Italy; concetta.castilletti@inmi.it (C.C.); chiara.agrati@inmi.it (C.A.); delia.goletti@inmi.it (D.G.); sara.leone@inmi.it (S.L.); alessandro.agresta@inmi.it (A.A.); eleonora.cimini@inmi.it (E.C.); eleonora.tartaglia@inmi.it (E.T.); rita.casetti@inmi.it (R.C.); francesca.colavita@inmi.it (F.C.); silvia.meschi@inmi.it (S.M.); giulia.matusali@inmi.it (G.M.); daniele.lapa@inmi.it (D.L.); saeid.najafi@inmi.it (S.N.F.); alessandra.aiello@inmi.it (A.A.); chiara.farroni@inmi.it (C.F.); paola.galli@inmi.it (P.G.); maria.capobianchi@inmi.it (M.R.C.); giuseppe.ippolito@inmi.it (G.I.)

**Keywords:** influenza, pneumoccocus, COVID-19, SARS-CoV-2, vaccine, immune response

## Abstract

Vaccination against SARS-CoV-2 is considered the most effective method of prevention to contain the pandemic. While highly effective SARS-CoV-2 vaccines are being applied on a large-scale, whether and to what extent the strength of the vaccine-induced immune response could be further potentiated is still an object of debate. Several reports studied the effect of different vaccines on the susceptibility and mortality of COVID-19, with conflicting results. We aimed to evaluate whether previous influenza and/or pneumococcal vaccination had an impact on the specific immune response to the SARS-CoV-2 BNT162b2 mRNA vaccine. The study population consists of 710 workers from our Institute who completed the BNT162b2 schedule and have been tested at least once after the second dose, from 27 December 2020 up to 15 April 2021. Of these, 152 (21.4%) had received an influenza and 215 (30.3%) a concomitant influenza and pneumococcal vaccination, a median of 102 days before the second dose of BNT162b2. Overall, 100% of workers were tested for anti-Spike receptor-binding domain (anti-S/RBD) antibodies, 224 workers for neutralization titer (Micro-neutralization assay, MNA), and 155 workers for a spike-specific T cell interferon-γ response (IFN-γ). The levels of anti-S/RBD, MNA and IFN-γ were evaluated and compared according to sex, age, involvement in direct care of COVID-19 patients, and previous influenza/pneumococcal vaccination. At the univariate analysis, no statistically significant association was observed with regard to a previous influenza and pneumococcal vaccination. A significant lower anti-S/RBD response was observed according to an older age and male sex, while MNA titers were significantly associated to sex but not to age. At the multivariable analysis, workers receiving a concomitant influenza and pneumococcal vaccination or only influenza showed a 58% (*p* 0.01) and 42% (*p* 0.07) increase in MNA titers, respectively, compared to those who did not receive an influenza/pneumococcal vaccination. Female workers showed an 81% MNA and a 44% anti-S/RBD increase compared to male workers (*p* < 0.001). Compared to workers aged 21 to 49 years, those aged 50 or older were associated with a reduction in the anti-S/RBD (16%; *p* 0.005), MNA (31%; *p* 0.019), and IFN.g (32%) immune response. Maintaining the influenza and pneumococcal immunization program for the coming season, in which COVID-19 could still be spreading, remains strongly recommended to protect those who are more vulnerable and to limit the potential burden of these infections on the healthcare system.

## 1. Introduction

Early after the beginning of the COVID-19 pandemic at the end of 2019, vaccination has been considered to be the most effective method of prevention, and in less than one year since the identification of the severe acute respiratory syndrome coronavirus-2 (SARS-CoV-2), an exceptional effort has led to the development of highly effective vaccines [1].

In particular, recently developed messenger RNA-based (mRNA) vaccines against SARS-CoV-2 have been rapidly approved for emergency use, and currently large-scale vaccination has started in many countries. Since mRNA vaccine technology is being applied for the first time on a large scale, several questions remain to be elucidated, and, among others, the question of whether and to what extent the strength of the vaccine-induced immune response could be further potentiated is still an object of debate [2].

Historically, vaccines have been known to have non-disease-specific effects that result in immunity to pathogens unrelated to the one specifically targeted, and that might influence the specific response to other unrelated vaccines [3,4].

No data are still available on the potential effect of influenza and pneumococcal vaccination on the response to a subsequent SARS-CoV-2 vaccination.

Therefore, we evaluated whether a previous influenza and/or pneumococcal vaccination had an impact on the specific immune response to an mRNA-based COVID-19 vaccine. Thus, we assessed the humoral- and cell-mediated immune response in the personnel of our Institute who completed the 2-dose BNT162b2 mRNA vaccine schedule, with the aim to evaluate the presence and to characterize the vaccination-induced B- and T-cell immune response among those who received the influenza/pneumococcal vaccination versus those who did not. 

## 2. Materials and Methods

### 2.1. Setting and Patient Selection

The National Institute for Infectious Diseases “L. Spallanzani” in Rome, Latium, has been the first Italian hospital to admit and manage patients affected by COVID-19 on 29 January 2020, with a dramatic increase in the number of admissions peaking to more than 200 in-patient daily presences during March and November 2020, and in March 2021, in accordance with the SARS-CoV-2 epidemic waves observed in Italy. 

On 27 December 2020, according to the Italian Ministry of Health recommendations, the Institute started a vaccination campaign against SARS-CoV-2 targeted to its staff. The BNT162b2 mRNA-based vaccine was the only one available at that time.

Since the beginning of the vaccination campaign, a study was implemented to follow up both the humoral and cell-mediated response to the BNT162b2. Following written informed consent, blood samples were collected at baseline (T0), just before (T1), and two weeks after the second dose (T2); demographic and occupational information were recorded. 

In the previous fall, from 7 October to 10 December 2020, all staff had been invited to receive both an influenza and pneumococcal vaccination. A quadrivalent cell-based inactivated influenza vaccine (Flucelvax [Seqirus, Inc., Maidenhead, UK]) and a 13-valent pneumococcal conjugate vaccine (PCV13) (Prevnar 13 [Pfizer Canada, Inc., Kirkland, QC, Canada]) or a pneumococcal polysaccharide vaccine (PPSV23) (Pneumovax 23 [Merck & Co., Inc., Kenilworth, NJ, USA]) were used.

Within the full cohort of personnel who received the BNT162b2 mRNA vaccine, we identified a convenience sample of subjects who have completed the 2-dose schedule and have been tested at T2 up to April 15. Subjects with a SARS-CoV-2 diagnosis, either because of scoring a real-time polymerase chain reaction (RT-PCR) positive to the molecular test on the nasopharyngeal swab, or because of being positive to anti-Nucleocapside (anti-N) and/or to anti-Spike receptor-binding domain (anti-S/RBD) antibodies at T0 or to anti-N at T1 or T2, were excluded. 

### 2.2. Laboratory Methods

Two commercial chemiluminescence microparticle antibody assays (CMIA), the SARS-CoV-2 specific anti-N and the anti-S/RBD tests (AdviseDx SARS-CoV-2 IgG II and SARS-CoV-2 IgG II Quant, respectively, ARCHITECT^®^ (Chicago, IL, USA) i2000sr Abbott Diagnostics, Chicago, IL, USA) were used according to the manufacturer’s instruction; Index >1.4 and Arbitrary units (AU)/mL >50 are considered positive, respectively.

A micro-neutralization assay (MNA) was performed as previously described, using SARS-CoV2/Human/ITA/PAVIA10734/2020 as the challenging virus [5]. Serum samples were heat-inactivated at 56 °C for 30 min and titrated in duplicate in seven two-fold serial dilutions (starting dilution 1:10). Equal volumes (50 μL) of serum and medium containing 100 TCID50 SARS-CoV-2 were mixed and incubated at 37 °C for 30 min. Serum-Virus mixtures were then added to sub-confluent Vero E6 cell monolayers and incubated at 37 °C and 5% CO_2_. After 48 h, microplates were observed by light microscope for the presence of CPE. The highest serum dilution inhibiting at least 90% of the CPE was indicated as the neutralization titer. To standardize inter-assay procedures, positive control samples showing a high (1:160) and low (1:40) neutralizing activity were included in each assay session. Serum from the National Institute for Biological Standards and Control, UK (NIBSC) with a known neutralization titer (research reagent for anti-SARS-CoV-2 Ab NIBSC code 20/130) was used as the reference in MNT. 

We studied IFN-γ responses as a surrogate of T-cell function. Peripheral blood was collected in heparin tubes and stimulated or not with a pool of peptides spanning the Spike protein (Miltenyi Biotech, Germany) at 37 °C (5% CO_2_). A superantigen (SEB) was used as the positive control. Plasma were harvested after 16–20 h of stimulation and stored at −80 °C. IFN-γ released in plasma after stimulation was quantified using an automated ELISA (ELLA, Protein Simple). The detection limit of these assays was 0.17 pg/mL.

### 2.3. Statistical Analysis

To identify possible factors associated with the strength of the anti-S/RBD, MNA, and IFN-γ immune response, the impact of age, sex, provision of direct care to COVID-19 patients, and previous influenza and/or pneumococcal vaccination was analyzed. 

Descriptive statistics were presented as a median with interquartile range (IQR) for continuous variables and a frequency with proportion for categorical variables.

Continuous variables were compared using a Wilcoxon signed-rank test or Kruskal–Wallis test followed by a post hoc Dunn’s test for pairwise multiple comparisons with a Bonferroni correction as appropriate.

A multiple linear regression was used to assess the association between the anti-S/RBD, MNA, and IFN-γ immune response and previous influenza/pneumococcal vaccination, adjusting for age, sex, and provision of direct care to COVID-19 patients. Since the distribution of data was positively skewed, a logarithmic transformation was performed to make the data conform more closely to the normal distribution and to improve the model fit.

A two-sided *p* value < 0.05 was considered statistically significant. Analyses were performed in R.

## 3. Results

The study population consisted of 710 workers. Out of them, 497 (70%) were women, with a median age of 43 years (IQR 31–52; range 21–75). Most were healthcare workers (*n* = 544, 77%) who cared directly for COVID-19 patients. Overall, 152 (21.4%) had only received an influenza vaccination and 215 (30.28%) a concomitant influenza and pneumococcal (207 PCV13 and 8 PPSV23) vaccination.

The median time from the influenza and pneumococcal vaccination to the second dose of SARS-CoV-2 vaccine was 102 days (range 42–160 d).

Overall, 100% of workers were tested for anti-S/RBD and presented a detectable response, with a median titer of 15,983.7 (IQR 9739.9; 24,331.1; range 125.2–447,049.3) AU/mL. In the subgroup of 224 workers for whom the MNA titer was performed, the median neutralizing activity was 1:80 (IQR 1:40–1:160; range 1:5–1: >640).

In the subgroup of 155 workers who had been monitored for the Spike-specific T cell response, the median of IFN-γ titers was 342.8 (IQR 189.1; 760.3; range 15.9–8874.0) pg/mL.

**Figure 1:**
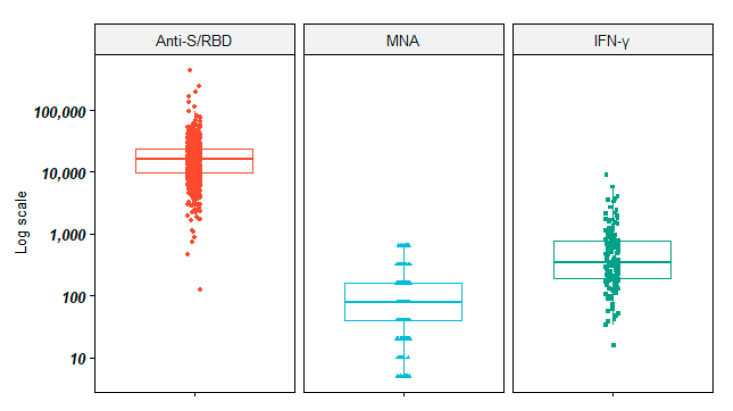
Overall distribution of anti-S/RBD, MNA, and IFN-γ titers elicited by BNT162b2. Legend: Anti-S/RBD: anti-Spike receptor-binding domain; MNA: Micro-neutralization assay titers; IFN: interferon. Bold line: median value.

Figure 1 shows the overall distribution of anti-S/RBD, MNA, and IFN-γ titers elicited by BNT162b2, and Table 1 summarizes the overall anti-S/RBD, MNA, and IFN-γ titers according to the listed variables.

At the univariate analysis, no statistically significant association was observed in the median titer of anti-S/RBD, MNA, and IFN-γ with regard to a previous influenza and pneumococcal vaccination.

In comparison to those workers who did not receive a influenza/pneumococcal vaccination, higher MNA median titers were observed in the group of workers who received only the influenza vaccination (*p* = 0.326) and both influenza and pneumococcal vaccinations (*p* 0.0736).

Anti-S/RBD titers were significantly lower in males (*p* = 0.0001) and in those aged 50 or older (*p* = 0.006). Additionally, there were statistically significant differences across the sexes in the MNA titers [*p* = 0.001].

**Figure 2:**
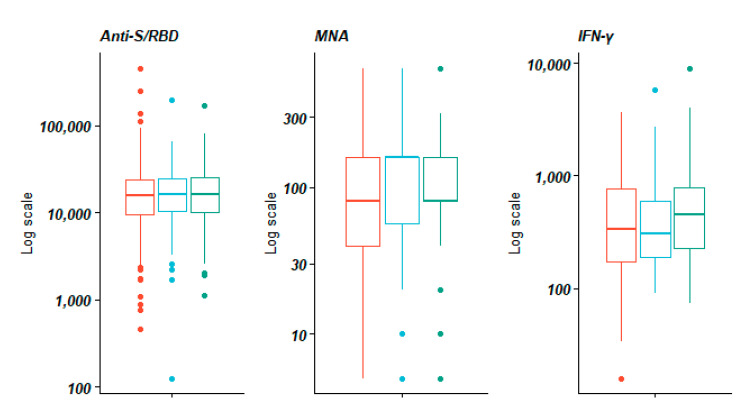
Title: Anti-S/RBD, MNA, and IFN-γ titers elicited by the BNT162b2 SARS-CoV-2 vaccine according to the influenza/pneumococcal vaccination. **Legend:** Anti-S/RBD: anti-Spike receptor-binding domain; MNA: Micro-neutralization assay titers; IFN: interferon Red: No influenza and pneumococcal vaccine; Light blue: Only influenza vaccine; Green: Influenza and pneumococcal vaccine. Bold line: median value.

Figure 2 shows the anti-S/RBD, MNA, and IFN- γ titers elicited by BNT162b2 according to the influenza/pneumococcal vaccination.

The results of the multiple regression model on the log-transformed anti-S/RBD, MNA, and IFN-γ titers are shown in Table 2.

After adjustment for sex, age group, and direct patient care, the model found that a previous concomitant influenza and pneumococcal vaccination was significantly associated with the MNA response to the BNT162b2, while no association was found between anti-S/RBD and IFN-γ with a previous influenza/pneumococcal vaccination. Namely, workers receiving a concomitant influenza and pneumococcal vaccination or only influenza showed a 58% (*p* 0.01) and 42% (*p* 0.07) increase in MNA titers, respectively, compared to those who did not receive a influenza/pneumococcal vaccination.

With regard to sex, female workers evidenced a 44% increase in anti-S/RBD titer (*p* < 0.01), and an 81% increase in MNA titers as compared to male workers (*p* < 0.001).

Compared to workers aged 21 to 49 years, workers aged 50 or older were associated with a significant reduction in all the values: a 16% reduction in anti-S/RBD titer (*p* 0.005), a 31% reduction in the MNA response (*p* 0.019), and a 32% reduction in the IFN-γ response (*p* = 0.046).

No statistically significant associations were found with respect to the workers’ role in COVID-19 patients’ care.

## 4. Discussion

Several reports have studied the effect of different vaccines on the susceptibility and mortality of COVID-19, with conflicting results [3,6,7,8,9,10,11,12].

A recent exploratory study of immunization records from 137,037 individuals diagnosed with SARS-CoV-2 positive PCR tests analyzed 18 different vaccines and found that most of them, including the PCV13 and influenza vaccine, administered in the past one, two, and five years were associated with decreased SARS-CoV-2 infection rates [3]. Moreover, the difference in mortality and symptom severity between children and elderly subjects in COVID-19 patients has been associated with the more recent vaccinations of children by a variety of vaccines, such as Bacillus Calmette–Guérin (BCG), diphtheria, pertussis and tetanus (DPT), hepatitis B, polio, rotavirus, and measles, mumps and rubella (MMR).

Other reports have studied the effect of influenza and pneumococcal vaccination on the susceptibility and mortality of COVID-19, with conflicting results.

An Italian study found that the influenza vaccine does not have a significant effect on COVID-19 hospitalization and mortality, except in those aged 65 years and older [10], and a recent ecological county-level study in the US showed a significant reduction in the COVID-19 mortality rate associated with a higher influenza vaccination coverage in the population aged 65 years and older [11].

Within a cohort of US adults aged ≥65 y, the receipt of PCV13 was associated with a lower incidence of any COVID-19 diagnosis, COVID-19 hospitalization, and fatal COVID-19 after correction for multiple potential sources of confounding, suggesting that protection arose from the prevention of early stages of COVID-19 pathogenesis rather than the prevention of severe post-infection sequelae. Indeed, the receipt of PPSV23—which, unlike PCV13, would not be expected to prevent pneumococcal colonization—showed little association with protection against COVID-19 outcomes [12].

The nonspecific innate response conferring protection of a vaccine to other infections has been termed ‘trained innate immunity”, an enhanced nonspecific immune response to an unrelated infection mediated by innate immune cells, such as monocytes, macrophages, and natural killer (NK) cells [4,13].

Instead, few data are available on whether vaccine-related trained innate immunity could also apply to the response to other vaccines [4].

In an experimental study conducted on healthy volunteers, BCG vaccination has been observed to enhance functional antibody responses against A(H1N1) influenza virus induced by an influenza vaccination 14 days subsequently. In the same study, the influenza vaccine exerted on its own enhanced responses to vaccination against certain unrelated pathogens but impaired responses against others [14].

In our real-world study, with respect to the main aim of our study, after adjustment for sex, age group, and direct patient care, we observed a substantial impact of the inactivated influenza vaccine alone or, more strongly, when associated to a pneumococcal conjugate/polysaccharide vaccination on the short term (i.e., two weeks after the second dose) neutralization response (MNA) elicited by the SARS-CoV-2 BNT162b2 mRNA vaccination completed a median of 102 days later.

In contrast, no statistically significant impact of the influenza/pneumococcal vaccination was observed on the anti-S/RBD, and IFN-γ response.

Of note, Khoury et al., basing themselves on evidence from clinical trials and convalescent cohort studies, recently suggested that neutralizing antibody levels were a highly predictive correlate of immune protection from symptomatic SARS-CoV-2 infection and an important predictor of vaccine efficacy [15].

As reported by others, we observed a strong positive association between female sex and humoral response [16], as well as a significant inverse relation between older age and humoral as well as cellular immune response [17].

It could be argued that an indication bias could be present in the study to confound the effect of influenza and pneumococcal vaccines due to the fact that both (especially the pneumococcal vaccine) could be more likely administered to older people. In turn, older people usually respond in a poorer way to any vaccination. However, the multiple regression analysis should have accounted for this potential bias.

The study was conducted in a single center and recruited healthy, young and middle-aged adults, predominantly female, without previous SARS-CoV-2 infection, and its findings should not be generalized to a different population. Moreover, we did not provide data on the possible impact of influenza and pneumococcal vaccination on the clinical effectiveness of the SARS-CoV-2 vaccine, and on the duration of immune protection after vaccination. Further prospective studies are needed to test our data and to explain the molecular bases of these results.

Despite the fact that in the last season influenza transmission and other infections transmitted mainly by droplets, have been significantly altered by the range of nonpharmaceutical interventions activated against COVID-19 [18,19], and the fact that a clear association relating Streptococcus pneumoniae infection to a COVID-19 outcome has not been defined [20], maintaining a broader eligibility of the influenza and pneumococcal immunization program for the coming season remains strongly recommended to protect those who are more vulnerable, as well as to limit the potential burden of these infections on the healthcare system [21].

## 5. Conclusions

Influenza/pneumococcal vaccination seems to have a substantial impact on the short term neutralization response, that is considered highly predictive of immune protection from symptomatic SARS-CoV-2 infection, to BNT162b2 mRNA vaccination completed few weeks later. This finding would be consistent with the observed protection of some vaccines on COVID-19 hospitalization and mortality, and suggests the need for further studies to test its reproducibility and to explain its molecular bases. While COVID-19 mass vaccination campaign is ongoing, influenza and pneumococcal immunization program for the coming season remains strongly recommended.

## Figures and Tables

**Table 1:** Anti-S/RBD, MNA, and IFN-γ titers to BNT162b2 SARS-CoV-2 vaccine, according to studied variables.

	antiS/RBD (AU/mL) Subjects Evaluated = 710		MNA (Neutralization Titre) Subjects Evaluated = 224		IFN-γ (pg/mL) Subjects Evaluated = 155	
Studied Variables	N.; (%)	Median [IQR]	N.; (%)	Median [IQR]	N.; (%)	Median [IQR]
Gender						
M	213; (30%)	12,180.5 [7708.1; 19,033.1]	75; (33.5%)	80 [40; 160]	45; (29%)	585.7 [190.5; 1223.7]
F	497; (70%)	17,085.9 [11,383.9; 26,391.4]	149; (66.5%)	80 [80; 160]	110; (71%)	322.4 [188.3; 673.9]
*p* value		*p* = 0.0001		*p* = 0.001		*p* = 0.068
Age						
21–49	468; (65.9%)	16,584.0 [10,542.9; 25,214.6]	91; (59.4%)	80 [40; 160]	105; (67.7%)	273.1 [190.5; 541.7]
>50	242; (34.1%)	13,531.7 [8687.9; 21,919.3]	133; (40.6%)	80 [40; 160]	50; (32.3%)	431.2 [188.3; 899.4]
*p* value		0.006		0.346		0.087
Direct care						
Yes	544; (76.6%)	16,002.2 [9740.1; 24,170.4]	166; (74.1%)	80 [40; 160]	127; (81.9%)	331.9 [182.4; 767.3]
No	166; (23.4%)	15,670.0 [9344.0; 24,331.1]	58; (25.9%)	120 [40; 320]	28; (18.1%)	365.9 [220.8; 707.5]
*p* value		0.692		0.155		0.691
Previous influenza/pneumococcal vaccinations						
1. No	343; (48.3%)	15,868.7 [9392.5; 23,685.0]	94; (42%)	80 [40; 160]	78; (50.3%)	332.6 [170.8; 765.0]
2. Influenza only	152; (21.4%)	16,203.5 [10,327.6; 24,695.6]	55; (24.6%)	160 [80; 160]	35; (22.6%)	307.1 [190.2; 591.5]
3. Concomitant Influenza and Pneumococcal	215; (30.3%)	15,994.7 [9925.0; 25,118.5]	75; (33.5%)	80 [80; 160]	42; (27.1%)	453.6 [222.0; 795.2]
*p* value		0.676		0.060		0.717
*p* value 1 vs. 2		1		0.326		1
*p* value 1 vs. 3		1		0.0736		1
*p* value 2 vs. 3		1		1		1

Legend: Anti-S/RBD: anti-Spike receptor-binding domain; MNA: micro-neutralization assay; IFN: interferon.

**Table 2:** Multiple regression with log-transformed anti-S/RBD, MNA, and IFN-γ titers elicited by BNT162b2 vaccine according to studied variables.

	Anti-S/RBD			MNA			IFN-γ		
Factors	RR	95% CI	*p* Value	RR	95% CI	*p* Value	RR	95% CI	*p* Value
Intercept	12,638.65	10,629.6, 15,027.4	<0.001	64.66	42.1, 99.31	<0.001	585.05	333.48, 1026.4	<0.001
Age group: ≥50 y	0.84	0.74, 0.95	0.005	0.69	0.51, 0.94	0.019	0.68	0.47, 0.99	0.046
Sex: Female	1.44	1.27, 1.63	<0.001	1.81	1.33, 2.48	<0.001	0.70	0.48, 1.02	0.064
Previous influenza/pneumococcal vaccinations: Influenza only	0.99	0.86, 1.15	0.928	1.42	0.97, 2.09	0.07	1.07	0.69, 1.67	0.756
Concomitant Influenza and Pneumococcal	1.08	0.95, 1.24	0.241	1.58	1.12, 2.23	0.01	1.21	0.81, 1.82	0.353
Direct patient care: Yes	0.93	0.81, 1.06	0.265	0.74	0.52, 1.04	0.08	0.86	0.54, 1.37	0.517

RR: Relative Risk. Anti-S/RBD: anti-Spike receptor-binding domain; MNA: micro-neutralization assay; IFN: interferon.

## Data Availability

Data available on request due to restrictions.
